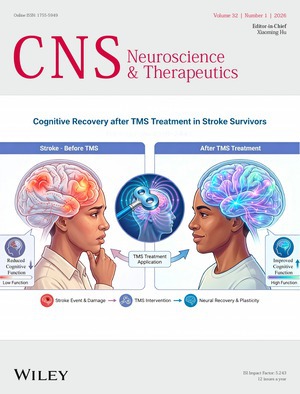# Front Cover

**DOI:** 10.1002/cns.70751

**Published:** 2026-01-19

## Abstract

Cover image: The cover image is based on the article *Recent Advances in Repetitive Transcranial Magnetic Stimulation for the Treatment of Post‐Stroke Cognitive Impairment* by Yu Liu et al., https://doi.org/10.1002/cns.70702.